# Salinity survival: molecular mechanisms and adaptive strategies in plants

**DOI:** 10.3389/fpls.2025.1527952

**Published:** 2025-02-28

**Authors:** Huankai Zhang, Caiyu Yu, Qian Zhang, Zihan Qiu, Xiansheng Zhang, Yifeng Hou, Jie Zang

**Affiliations:** ^1^ College of Life Sciences, Zaozhuang University, Zaozhuang, China; ^2^ State Key Laboratory of Wheat Improvement, Peking University Institute of Advanced Agricultural Sciences, Shandong Laboratory of Advanced Agricultural Sciences in Weifang, Weifang, Shandong, China; ^3^ State Key Laboratory of Wheat Improvement, College of Life Sciences, Shandong Agricultural University, Tai’an, Shandong, China

**Keywords:** salt stress, molecular design breeding, crop production, Na^+^, molecular mechanisms

## Abstract

Soil salinity is a significant environmental challenge that threatens plant growth and development, adversely affecting global food crop production. This underscores the critical need to elucidate the molecular mechanisms underlying plant salt tolerance, which has profound implications for agricultural advancement. Recent progress in plant salt tolerance has greatly improved our understanding of the molecular mechanisms of plant responses to salt stress and precision design breeding as an effective strategy for developing new salt-tolerant crop varieties. This review focuses on the model plant species *Arabidopsis thaliana* and important crops, namely, wheat (*Triticum aestivum*), maize (*Zea mays*), and rice (*Oryza sativa*). It summarizes current knowledge on plant salt tolerance, emphasizing key aspects such as the perception and response to salt stress, Na^+^ transport, Na^+^ compartmentalization and clearance, changes in reactive oxygen species induced by salt stress, and regulation of plant stem cell development under salt stress conditions. The review might provide new and valuable information for understanding the molecular mechanisms of plant response and adaptation to salt stress.

## Introduction

Plants, as sessile organisms, face numerous abiotic stresses throughout their life cycle, including drought, salinity, temperature fluctuations, heavy metal ion exposure, ultraviolet radiation, and other physical disturbances (Sun H. et al., 2020; Markham and Greenham, 2021; Zhang et al., 2022a; Kopecka et al., 2023). These stresses not only influence the geographical distribution of plants but also significantly affect their growth, development, and agricultural productivity (Villalobos-Lopez et al., 2022; Zhang et al., 2023a).

Among these challenges, salt stress is particularly critical, with soil salinization posing a major environmental threat to global crop sustainability. During crop domestication, their tolerance to abiotic stress has typically diminished. Soil salinity currently impacts approximately 7% of the world’s land area, covering approximately 950 million hectares (Munns and Tester, 2008; Yang and Guo, 2018a). Of the 230 million hectares of irrigated land globally, 20% to 30% are affected by various degrees of salinization, a figure that continues to grow due to land clearance and agricultural irrigation (Ismail and Horie, 2017; Liang et al., 2024). Projections indicate that global climate change will increase the frequency, duration, and severity of extreme weather events such as droughts and heat waves (Pareek et al., 2020a, b). Prolonged droughts and high temperatures have driven the expansion of irrigation systems worldwide, further exacerbating land salinization. Currently, 52% of the global population, across 13 countries, faces severe salinization issues (Liu M. et al., 2020). As freshwater resources dwindle, agriculture in the 21st century is increasingly contending with saline conditions (Rawat et al., 2022).

**Figure 1:**
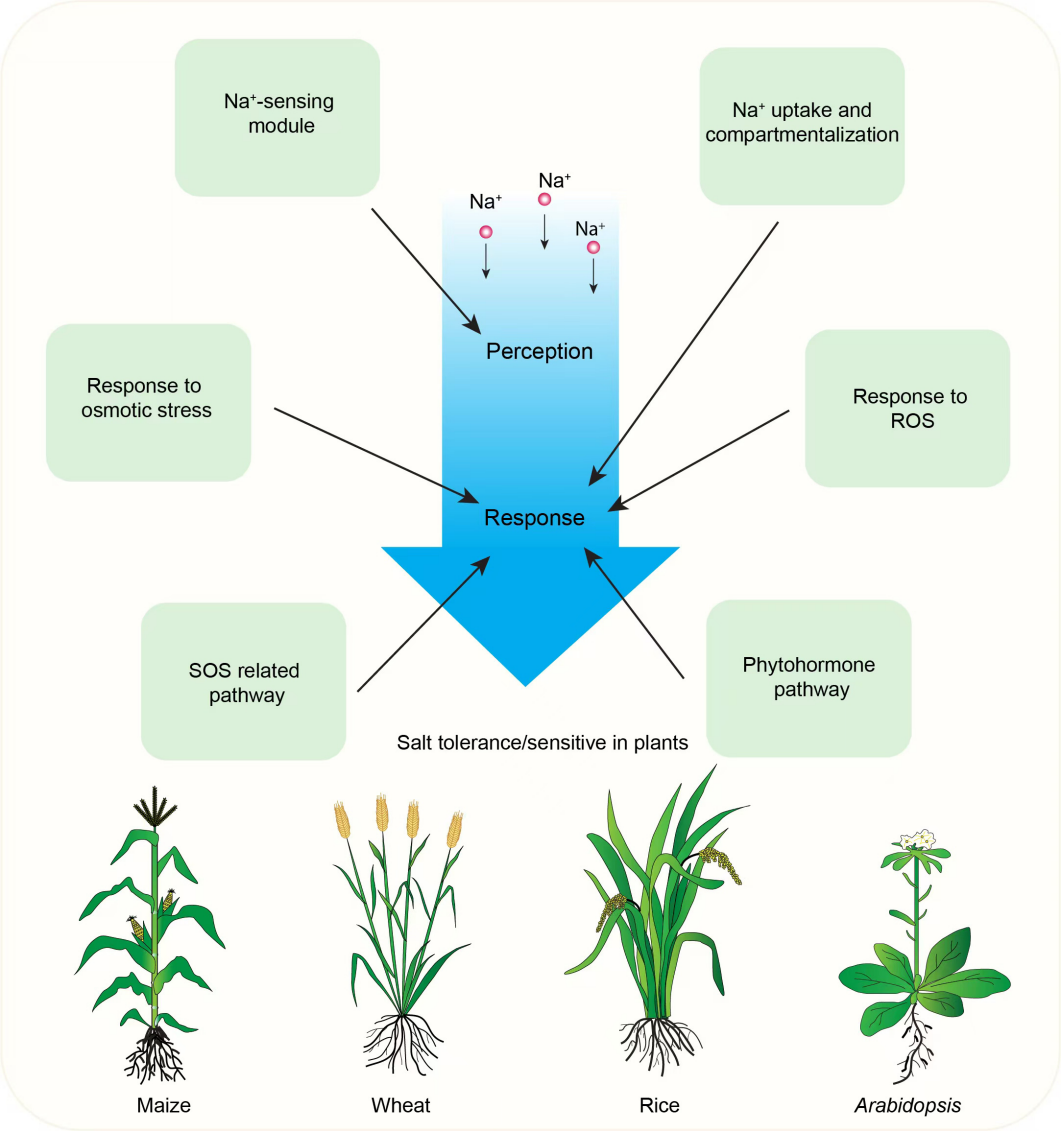
Illustration of plant salt stress responses and mechanisms. Green boxes represent key aspects of plant responses to salt stress, including the processes of salt ion perception and response. The salt stress perception process involves a well-characterized Na^+^-sensing module. Key mechanisms depicted include the SOS pathway, Na^+^ uptake and compartmentalization, osmotic stress response, reactive oxygen species response, and phytohormone signaling pathways.

Despite the challenges posed by environmental factors like soil salinization, the global population is projected to reach nearly 10 billion by 2050 (Bailey-Serres et al., 2019; Hickey et al., 2019), driving a 60% increase in food demand. At the same time, urbanization is rapidly reducing the available arable land. In combination with drastic environmental changes, key food crops, such as wheat, rice, and maize, are particularly sensitive to salinity. Therefore, crop breeders must focus on developing high-yielding, salt-tolerant varieties to improve productivity on salt-affected land and expand cultivable areas. This strategy is essential to address climate change and ensure global food security. In this review, we focus on summarizing our current understanding of plant salt tolerance mechanisms. We discuss plant perception and response to salt stress; Na^+^ transport, compartmentalization, and clearance; reactive oxygen species (ROS) changes induced by salt stress; and regulation of plant stem cell development under saline conditions (**Figure 1**). Finally, we explore future challenges and opportunities in understanding crop salt tolerance mechanisms and breeding new, high-yielding, and salt-tolerant varieties.

## Salt stress perception in plants

Under high-salt stress conditions, plants have evolved a range of complex physiological responses to adapt and resist these adverse environments. This suggests that plant roots possess Na^+^ receptors, enabling salt-avoidance growth strategies (Sun et al., 2008; Galvan-Ampudia et al., 2013; Wang et al., 2022b). When exposed to high salinity, plants must contend not only with osmotic stress but also with the ionic stress caused by Na^+^ ions. Therefore, Na^+^ uptake can potentially contribute to ionic stress. These ionic stresses are often accompanied by tissue-specific Ca²^+^ oscillations and amplitude changes within the Na^+^ signaling pathway (Knight et al., 1997; Ma et al., 2019; Steinhorst et al., 2022). The *Monocation-Induced [Ca^2+^]_i_ Increases1* (*MOCA1*) gene encodes a glucuronyl transferase responsible for adding a negatively charged glucuronic acid (GlcA) group to inositol phosphorylated ceramide (IPC), thereby generating glycosyl inositol phosphorylated ceramide (GIPC) sphingolipids. These GIPC sphingolipids bind Na^+^ cations, causing cell membrane depolarization and salt-dependent intracellular Ca²^+^ oscillations. However, the specific Ca²^+^ channels involved in this reaction remain unclear (Cao et al., 2019; Jiang et al., 2019).

Studies show that MOCA1-dependent GIPC functions as a sensor for environmental Na^+^ fluctuations, involving Ca²^+^ transporters. In *Arabidopsis*, two highly relevant Ca²^+^ permeable transporters, ANNEXIN1 (AtANN1) and AtANN4, are crucial in responding to salt-induced Ca²^+^ signals (Laohavisit et al., 2013; Ma et al., 2019). AtANN1 is essential for promoting salt-activated Ca²^+^ influx in the plasma membranes of root epidermal cells, while AtANN4 plays a pivotal role in increasing salt-induced [Ca²^+^]_cyt_ and activating the Salt Overly Sensitive (SOS) pathway (Ma et al., 2019). AtANN4 is regulated by SOS2 protein phosphorylation, and in the presence of its interacting protein SCaBP8 (also known as CBL10), it modulates salt-induced Ca²^+^ signaling through a phosphorylation-dependent negative feedback loop (Ma et al., 2019).

Salt stress not only disrupts the plasma membrane but also negatively affects multiple organelles within plant cells. Each organelle must be capable of sensing and responding to salt stress signals (Zhao et al., 2020). In *Arabidopsis*, the LRX-RALF-FER module—including Cell Wall Leucine-Rich Repeat Extensins 3/4/5 (LRX3/4/5), Rapid Alkalinization Factor 22/23 (RALF22/23), and Receptor-Like Kinase FERONIA (FER)—plays a key role in sensing salt stress and regulating salt tolerance (Zhu, 2016; Byrt et al., 2018; Feng et al., 2018; Zhao et al., 2018, 2020). This module senses changes in the cell wall during salt stress, such as the displacement of pectin-cross-linked Ca²^+^ and the accumulation of reactive oxygen species (ROS), triggering signal transduction pathways that maintain cell wall integrity (CWI) and prevent cell damage (Feng et al., 2018; Zhao et al., 2020).

LRX3/4/5 proteins, located in the cell wall, regulate CWI by interacting with RALF22/23 peptides, preventing their binding to FER under normal conditions. However, under salt stress, the mature RALF22/23 proteins are released and interact with FER, inducing its internalization via an endosomal pathway (Zhao et al., 2018). Mutants such as *lrx 345* and *fer-4*, as well as plants overexpressing *RALF22/23*, exhibit slow growth and heightened sensitivity to salt stress (Zhao et al., 2018, 2021). Therefore, the LRX3/4/5–RALF22/23–FER module plays a critical role in regulating plant growth, maintaining CWI, and responding to salt stress.

The *Arabidopsis* TZF1 protein, along with the rice proteins OsBAG6 and OsCaM1-1, plays essential roles in plant perception and response to salt stress (He et al., 2024; Wang et al., 2024b). The *Arabidopsis* tandem CCCH zinc finger (TZF) protein, TZF1, binds to and promotes the degradation of autoinhibited Ca^2+^-ATPase 11 (ACA11) mRNA, enhancing salt tolerance. ACA11 encodes a tonoplast-localized calcium pump responsible for exporting calcium, thereby modulating key signal transduction pathways critical for salt tolerance (He et al., 2024). In rice, the mitochondrial-localized chaperone regulator OsBAG6, a member of the B-cell lymphoma 2 (Bcl-2)-associated athanogene family, acts as a novel negative regulator of salt-alkali stress tolerance. Loss-of-function mutations in *osbag6* reduce sensitivity to salt-alkali stress. Under non-stress conditions, OsBAG6 binds to the calcium sensor OsCaM1-1, but as Ca²^+^ levels rise, OsBAG6 releases OsCaM1-1, allowing the Ca²^+^-saturated OsCaM1-1 to regulate downstream stress-responsive genes as part of the salt-alkali stress response (Wang et al., 2024b). Additionally, functional variants in ZmCBL8 (Calcineurin B-like protein), a component of the Salt Overly Sensitive pathway, have been found to confer salt tolerance in maize (Wang et al., 2024d). In addition, recent studies suggest that cellulose and β-1,4-galactan have biological functions in regulating plant salt tolerance (Zhang et al., 2016b; Yan J. et al., 2021; Yan J. et al., 2023). Although considerable progress has been made in identifying genes and signaling pathways related to plant salt perception, the precise mechanisms through which plants sense salt ions, including the intracellular Na^+^ receptors, remain largely unresolved.

## Plant responses to salt stress

### Plant responses to salt‐induced osmotic stress

Plants respond to high-salt environments by altering their osmotic potential. Over time, they have evolved a range of sensing and response mechanisms that regulate osmotic stress signals induced by salt stress (Christmann et al., 2013; Shabala et al., 2016; Gong et al., 2020; Wang et al., 2022b; Zhang et al., 2023a). Among these mechanisms, Ca²^+^ functions as a crucial secondary messenger in sensing and signaling under various abiotic stresses (Gong et al., 2020; Dong et al., 2022; Zhang et al., 2022a). In plants, the *Reduced Hyperosmolality-Induced [Ca^2+^]_i_ Increase1* (*OSCA1*) gene encodes a hyperosmolarity-gated calcium channel that acts as an osmotic stress sensor. This channel detects rapid increases in cytosolic Ca²^+^ ([Ca²^+^]_cyt_) levels triggered by osmotic stress (Knight et al., 1997; Yuan et al., 2014). In *osca1* mutants, Ca²^+^ influx under high osmolarity is impaired, leading to reduced leaf transpiration and hindered root growth (Jojoa-Cruz et al., 2018; Liu et al., 2018; Maity et al., 2019). Similarly, in *Arabidopsis thaliana*, the *MSCS-LIKE 8* (*MSL8*) gene encodes a membrane tension-gated ion channel that responds to osmotic stress by upregulating expression during low osmotic conditions. This process facilitates ion efflux, helping protect cells from hypoosmotic stress (Hamilton et al., 2015). These genes are central to osmotic stress sensing in plants.

Recent research has revealed that Ca²^+^-responsive proteins such as BONZAI (Chen et al., 2020a), plastid K^+^ exchange antiporters (Stephan et al., 2016), small-conductance mechanosensitive ion channel-like channels (Hamilton et al., 2015), and MID1-COMPLEMENTING ACTIVITY (MCA) channels (Kurusu et al., 2012a, b) activate downstream pathways in response to osmotic stress-induced changes in cell turgor pressure. Furthermore, SEUSS, a transcriptional co-regulator of AGAMOUS, plays a critical role in osmotic stress responses. Under hyperosmotic stress, SEUSS forms liquid-like nuclear condensates, which are associated with osmotic stress tolerance and gene expression regulation. The absence of SEUSS significantly impairs the expression of osmotic stress tolerance genes (Wang et al., 2022a).

Abscisic acid (ABA), a key plant hormone, regulates various physiological processes that allow plants to survive under adverse environmental conditions. The role of ABA in plant adaptation to drought, cold, and salinity stress is well established (Zhu, 2016). Salt-induced osmotic stress triggers the expression of ABA biosynthesis genes, including *NINECIS-EPOXYCAROTENOID DIOXYGENASEs* (*NCEDs*), *ABA DEFICIENT* (*ABA*), and *ABA Aldehyde Oxidase* (*AAO3*), promoting ABA accumulation and enhancing environmental adaptability (van Zelm et al., 2020; Du et al., 2023). Protein phosphorylation is key to ABA signal transduction under stress conditions. Conserved in *A. thaliana*, rice, and maize, clade A-type 2C protein phosphatases (PP2Cs) and subclass III SNF1-related protein kinase 2s (SnRK2s) are central to this process (Geiger et al., 2009; Sirichandra et al., 2009; Klingler et al., 2010; Sun et al., 2016; Min et al., 2019; Wu et al., 2019; Wang et al., 2020c). Salt-induced ABA accumulation is detected by the Pyrabactin Resistance 1/PYR1-Like/Regulatory Component of ABA Receptors (PYR/PYL/RCAR) family, which inhibits PP2Cs, leading to the release of SnRK2s. These SnRK2s phosphorylate downstream anion efflux channels and transcription factors, regulating stomatal closure and gene expression (Negi et al., 2008; Chen et al., 2020b; Takahashi et al., 2020). This phosphorylation activates ABA-responsive element (ABRE)/ABRE-binding factor transcription factors, which promote the expression of stress-responsive genes like β-AMYLASE1 and α-AMYLASE, contribute to osmolyte accumulation, and improve water and nutrient uptake (Thalmann et al., 2016).

ABA is synthesized in various plant tissues, including vascular tissues and guard cells, and is transported to long distances to ensure proper signal transduction (Christmann et al., 2005; Li et al., 2018). For instance, under dehydration stress, the CLAVATA3/Embryo-Surrounding Region-Related 25 (CLE25) peptide is induced in the roots and transported to the leaves via the vascular system, where it triggers ABA biosynthesis and accumulation, promoting stomatal closure (Takahashi et al., 2018). *CLE25* loss-of-function mutants exhibit heightened sensitivity to salt stress (Takahashi et al., 2018).

In addition to PP2Cs and SnRK2s, mitogen-activated protein kinase (MAPK) cascades, composed of MAP kinase kinase kinases (MKKKs/MAP3Ks/MEKKs), MAP kinase kinases (MKKs/MAP2Ks/MEKs), and MAP kinases (MAPKs/MPKs), are pivotal in plant responses to osmotic stress (Gasulla et al., 2016). These MAPK cascades exhibit functional diversity, allowing plants to adapt to various environmental stimuli and internal demands (Zhang et al., 2022a). In *Arabidopsis*, the MKK4-MPK3 and MKKK20-MPK6 modules are vital in osmotic stress responses, and loss-of-function mutations in MKKs improve tolerance to dehydration and salinity (Kim et al., 2011, 2012). The bread wheat TMPK3 (wheat Mitogen-Activated Protein Kinase) plays a vital role in plant tolerance to salt and osmotic stresses. TMPK3 can autophosphorylate *in vitro* and is phosphorylated by the constitutively active *Arabidopsis* kinase AtMKK2. Additionally, TMPK3 phosphorylates its substrate, MAP kinase phosphatase 1. Notably, TMPK3 can compensate for the salt sensitivity in the *Arabidopsis mpk3-1* loss-of-function mutant, and its overexpression significantly enhances plant tolerance to salt and osmotic stresses beyond the levels observed in wild-type plants (Ghorbel et al., 2023). Similarly, Raf-like protein kinases (RAFs), which act as MKKKs, are essential for ABA-triggered SnRK2 activation under salt-induced osmotic stress (Lin et al., 2021).

Accumulation of metabolic substances like proline, hydroxyproline, glycine betaine, sugars, and polyamines helps stabilize osmotic pressure changes under salt stress. These osmolytes mitigate water loss and improve cell turgor (Pommerrenig et al., 2007; Henry et al., 2015; Mansour and Ali, 2017). Salt-induced proline accumulation maintains H3K4me3 levels at the Δ1-PYRROLINE-5-CARBOXYLATE SYNTHETASE 1 locus (Feng et al., 2016). A metabolomics analysis of 266 maize inbred lines identified 37 metabolic biomarkers (METOs) associated with salt-induced osmotic stress (SIOS). Research further revealed that ZmCS3 (citrate synthase), ZmUGT (glucosyltransferase), and ZmCYP709B2 (cytochrome P450) contribute to METO-SIOS tolerance (Liang et al., 2021).

Multiple studies suggest that salt stress impacts plants through osmotic stress, to which they have developed various adaptive mechanisms. These include Ca²^+^ signaling, ABA pathways, protein phosphorylation, kinase cascades, and osmolyte accumulation.

### Salt stress tolerance and Na^+^ transport in plants

**Figure 2:**
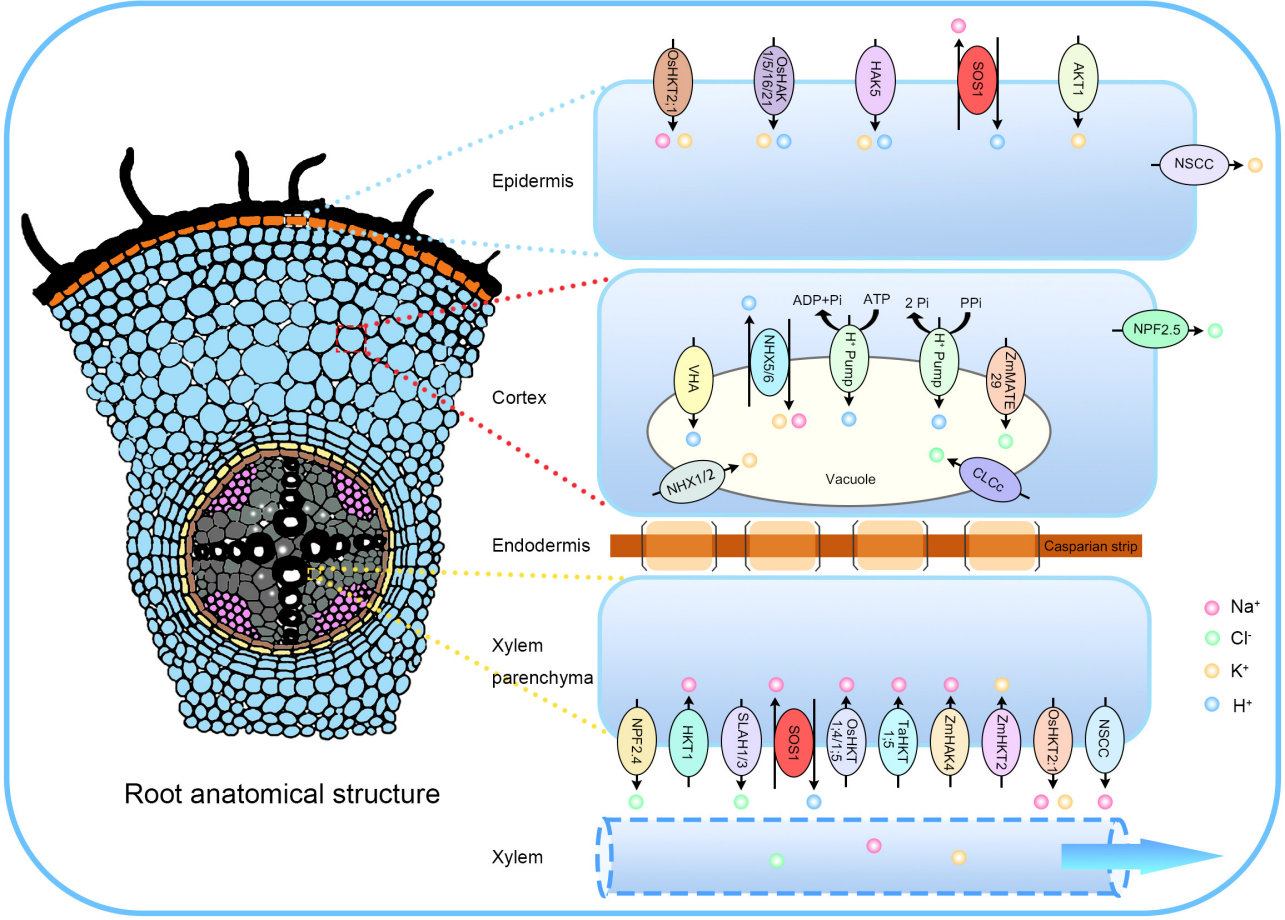
The translocation and compartmentalization of Na^+^, K^+^, and Cl^−^ facilitated by transporters. Salt ions move symplastically across the epidermis, cortex, and endodermis, entering the xylem via xylem parenchyma cells. Na^+^ enters root cells through the plasma membrane (PM) via Ca²^+^-permeable non-selective cation channels (NSCCs) and the high-affinity K^+^ transporter OsHKT2;1 in rice. The SOS1 protein mediates Na^+^ extrusion, while K^+^-permeable NSCCs contribute to salt-induced K^+^ loss. To maintain K^+^ homeostasis, the influx of K^+^ is facilitated by PM inward K^+^ channels, including HAK5 and AKT1. Within cortical cells, NPF2.5 removes Cl^−^ from the roots, while ZmMATE29 sequesters Cl^−^ into vacuoles. Na^+^ sequestration into root vacuoles is mediated by the antiporter NHX, while Cl^−^ sequestration occurs through the chloride channel CLCc, which operates with energy from H^+^-ATPase or H^+^-pyrophosphatase (H^+^-PPase) pumps. The Casparian strip prevents apoplastic entry of Na^+^ and Cl^−^ into the stele. In xylem parenchyma cells, passive Na^+^ loading occurs via NSCCs and OsHKT2;1, while active Na^+^ loading is mediated by SOS1. Transporters such as HKT1, OsHKT1;4, OsHKT1;5, TaHKT1;5, and ZmHAK4 are involved in the retrieval of Na^+^ from the root xylem to distribute Na^+^ between the root and shoot. OsHKT2;1 is responsible for loading K^+^ into the xylem, while ZmHKT2 removes K^+^ from the xylem. Finally, NPF2.4, SLAH1, and SLAH3 are key facilitators of Cl^−^ transport into the root xylem.

Plants exposed to high-salt environments face two primary types of stress that are exposed to high-salt environments: osmotic stress, caused by changes in osmotic potential that reduce water absorption, and ionic toxicity, due to the accumulation of toxic ions such as Na^+^. While osmotic stress has been extensively reviewed, the study of Na^+^ transport and accumulation is equally critical for understanding salt stress in plants. As sessile organisms, plants rely primarily on their roots to absorb water, nutrients, and ions from their surroundings. In high-salt environments, Na^+^, the most abundant soluble cation, enters plants through various ion channels and transporters in the roots, including calcium-permeable non-selective cation channels (NSCCs) and high-affinity K^+^ transporters (HKTs) (Tester and Davenport, 2003; Mian et al., 2011) (**Figure 2**).

Transpiration leads to significant water loss, which in turn increases the concentration of ions like Na^+^ within plant tissues. To combat this, plants have developed sophisticated mechanisms to exclude excess salts from their cells (Munns et al., 2020). Studies have shown that plants primarily rely on membrane transporters responsible for Na^+^ uptake, export, and compartmentalization to limit Na^+^ accumulation, thereby mitigating salt stress. These mechanisms are vital for maintaining intracellular ion homeostasis and preserving normal physiological functions in salt-stressed plants (Gong et al., 2020; Liang et al., 2024). In recent years, an increasing number of studies have reported that membrane transporters and membrane proteins play a crucial role in the salt tolerance of plants (Isayenkov and Maathuis, 2019; Banik and Dutta, 2023; Dutta, 2023; Mishra et al., 2024). While these processes have been extensively studied in *A. thaliana*, they have also been observed in a variety of crop species, highlighting their broader relevance.

### Na^+^ uptake

Roots serve as the primary organs for Na^+^ absorption in plants. The ion traverses the root epidermis, cortex, and endodermis before entering the xylem, where it is transported to the stem tissue. Excessive accumulation of Na^+^ in the stem can lead to toxic effects, prompting plants to evolve various mechanisms to limit Na^+^ entry into the root xylem and facilitate its recovery, thereby reducing transport to the stem.

HKT-type transporter proteins play a pivotal role in the retrieval of Na^+^ from the root xylem, significantly contributing to plant salt tolerance (Gong et al., 2020). Members of the HKT1 subfamily are known to reclaim Na^+^ from the root xylem, thereby minimizing its transport from roots to stems. This includes AtHKT1 in *Arabidopsis*; OsHKT1;1, OsHKT1;4, and OsHKT1;5 in rice; TaHKT1;4 and TaHKT1;5 in wheat; and ZmHKT1;1 and ZmHKT1;2 in maize (Ren et al., 2005; Huang et al., 2006; Munns et al., 2012; Byrt et al., 2014; Campbell et al., 2017; Oda et al., 2018; Zhang et al., 2018, 2023b; Wang et al., 2024c). In *Arabidopsis*, the *athkt1* mutants exhibit excessive Na^+^ accumulation and hypersensitivity to Na^+^ in the stem (Berthomieu et al., 2003). *AtHKT1* functions in root parenchyma cells to retrieve Na^+^ from the root xylem, thereby mitigating Na^+^ accumulation in the stem. Specifically, overexpression of *AtHKT1* in the root stele enhances salt tolerance in *Arabidopsis* (Moller et al., 2009). Under salt stress, PP2C49, a type G PP2C, inhibits Na^+^ permeability of AtHKT1 (Chu et al., 2021).

In rice, *OsHKT1;5* is crucial for removing Na^+^ from the root xylem, thus conferring salt tolerance to the plant (Ren et al., 2005; Kobayashi et al., 2017). Salt stress enhances the interaction between OsDNAJ15 and OsBAG4 in the nucleus, promoting the formation of transcriptional complexes that activate *OsHKT1;5* (Liu et al., 2023). Additionally, the loss of function of OsWRKY53 facilitates Na^+^ homeostasis mediated by OsMKK10.2 and OsHKT1;5 (Yu J. et al., 2023). Literature also suggests that epigenetic modifications are involved in gene expression mediated by AtHKT1 and OsHKT1;5 under salt stress (Baek et al., 2011; Wang et al., 2020a; Liu et al., 2022a, b). Mutations in *OsHKT1;1* can affect Na^+^ accumulation in rice roots and alter salt tolerance (Campbell et al., 2017).

In wheat, *TaHKT1;4* and *TaHKT1;5* are major genes that reduce Na^+^ transport and accumulation, enhancing salt tolerance. *TaHKT1;4* sequesters Na^+^ in the leaf sheath, minimizing its transport and accumulation in leaf blades (Huang et al., 2006; James et al., 2006). Conversely, TaHKT1;5 functions similarly to AtHKT1, facilitating the retrieval of Na^+^ from the root xylem (Byrt et al., 2007). The SPL6-HKT1;5 module offers a target for the molecular breeding of salt-tolerant crops (Wang et al., 2024c). Notably, *TaHKT1;5* enables durum wheat to increase grain yield by 25% in saline soils, significantly enhancing its economic value for developing salt-tolerant, high-yielding crop varieties (Munns et al., 2012).

In maize, the functions of *ZmHKT1;1* and *ZmHKT1;2* are closely linked to salt tolerance. Mutations in *ZmHKT1;1* result in increased Na^+^ content in the leaves and a salt-sensitive phenotype (Zhang et al., 2018). ZmHKT1;2 is also associated with Na^+^ content in the shoots of maize seedlings under salt stress (Zhang et al., 2019, 2023b). Modern research indicates that salt tolerance in maize is a complex trait governed by multiple genes that influence Na^+^ transport and distribution within the plant.

The HKT1 subfamily of transporters primarily mediates sodium ion transport. In contrast, the HKT2 subfamily, exclusive to monocotyledonous plants, uniquely transports both sodium and potassium ions (Hamamoto et al., 2015; Ali et al., 2019). However, HKT2 transporters can also mediate Na^+^ uptake. For instance, *ZmNC2*/*ZmHAK4* functions as a Na^+^-selective transporter located in the xylem parenchyma cells of the root stele, reducing Na^+^ accumulation in the stem by facilitating Na^+^ removal from the root xylem (Zhang et al., 2019). In rice, OsHKT2;1, localized to the plasma membrane, mediates Na^+^ uptake but is inhibited under salt stress (Horie et al., 2007). *OsHAK12* promotes Na^+^ exclusion from stem tissues via a mechanism akin to *ZmHAK4* (**Figure 2**) (Zhang et al., 2021).

Salt stress also promotes the development of the Casparian strip (CS), which forms a barrier that prevents salt ions from entering the root stele through the apoplastic pathway (Chen et al., 2011; Wang et al., 2022c). In summary, by minimizing the transport of Na^+^ from roots to shoots and enhancing its retrieval from the root xylem, plants can effectively mitigate the toxic effects of salt stress.

### Functional analysis of the SOS signaling pathway in mediating root Na^+^ efflux

The discovery of SOS genes has provided a foundational understanding of plant salt tolerance. Over the past few decades, scientists have increasingly recognized the central role of the SOS signaling pathway in mediating Na^+^ extrusion from plant roots (Shi et al., 2002; Yang and Guo, 2018b). Through mutant screening experiments, researchers have identified various salt-sensitive mutants (*sos* mutants), cloned SOS genes, and thoroughly characterized their functions. It is now widely accepted that the SOS signaling pathway comprises three key proteins: SOS1, SOS2, and SOS3 (Zhu, 2001).

The *SOS1* gene is highly conserved across sequenced genomes and encodes a plasma membrane Na^+^/H^+^ antiporter that utilizes the H^+^ gradient to drive Na^+^ extrusion, thereby lowering cytoplasmic Na^+^ concentrations (**Figure 2**) (Shi et al., 2000, 2002, 2003; Steinhorst et al., 2022; Zhang et al., 2023c, d). In *A. thaliana*, SOS1 uniquely possesses a long cytoplasmic C-terminal region exceeding 700 amino acid residues (Shi et al., 2000). This C-terminal region contains a self-inhibitory domain that interacts with upstream sequences harboring putative cyclic nucleotide monophosphate (cNMP) binding motifs, inhibiting SOS1 transport activity under normal conditions. These motifs are essential for SOS1 function, and mutations in these motifs lead to its inactivation (Shi et al., 2000). This suggests that SOS1 may be regulated by cyclic nucleotides, signaling molecules that mediate plant environmental adaptation and play roles in responses to salt and osmotic stress (Shabala et al., 2015; Swiezawska et al., 2018). The C-terminal region also interacts with numerous regulatory proteins and undergoes phosphorylation, modulating SOS1 antiporter activity (Fliegel, 2019). Under high-salt conditions, serine residues within this self-inhibitory domain are phosphorylated by SOS2, activating SOS1 to transport Na^+^ out of the cell and prevent salt toxicity (Quintero et al., 2011). The *SOS2* gene encodes a member of the SNF1-related kinase 3 (SnRK3s), also known as *CBL-interacting protein kinase 24* (*CIPK24*), which comprises 25 members in *A. thaliana*. Under high-salt conditions, the autoinhibition of SOS2 is relieved, enabling it to activate SOS1 through phosphorylation (Liu et al., 2000; Guo et al., 2001; Quintero et al., 2002). Meanwhile, *SOS3* (also known as *CBL4*) encodes a Ca²^+^-binding protein with three EF-hand motifs that senses the elevation of Ca²^+^ levels triggered by salt stress. Its family includes 10 members (Liu and Zhu, 1998; Ishitani et al., 2000; Kim et al., 2007; Quan et al., 2007; Steinhorst et al., 2022; Yang and Guo, 2018a; Zhu, 2016).

The SOS3–SOS2–SOS1 module plays a critical role in plant salt tolerance. Mutants lacking any of these three genes exhibit heightened sensitivity to NaCl, underscoring their essential function in conferring salt tolerance. In this signaling pathway, SOS3 detects the salt-induced rise in Ca²^+^ levels and binds to the autoinhibitory domain of SOS2, activating it. The N-terminus of SOS3 is myristoylated, allowing it to bind to the plasma membrane and recruit the SOS3–SOS2 complex, where SOS2 phosphorylates SOS1. This phosphorylation enhances the Na^+^/H^+^ antiporter activity of SOS1, promoting Na^+^ extrusion and lowering intracellular Na^+^ concentrations. The Na^+^ extrusion mediated by the SOS3–SOS2–SOS1 module is a critical mechanism for maintaining ion homeostasis and preventing salt toxicity under high-salt conditions (Halfter et al., 2000; Qiu et al., 2002; Kim et al., 2007; Quan et al., 2007; Quintero et al., 2011; Steinhorst et al., 2022; Zhang et al., 2023c, d).

The CBL–CIPK signaling pathway plays a pivotal role in plant responses to various abiotic stresses (Weinl and Kudla, 2009; Kudla et al., 2018). Under salt stress, SOS2 specifically interacts with and phosphorylates SCaBP8 (also known as CBL10), promoting the recruitment of SOS2 to the plasma membrane, where SOS2 then phosphorylates SOS1, enhancing its Na^+^ extrusion transporter activity (Quan et al., 2007; Lin et al., 2009). Additionally, the SOS2–SCaBP8 complex phosphorylates and inhibits the putative Ca²^+^-permeable transporter AtANN4, forming a negative feedback loop to fine-tune Ca²^+^ signaling in response to salt stress (Ma et al., 2019). Research indicates that the SOS2–SOS3 complex primarily functions in the roots, while the SOS2–SCaBP8 complex acts predominantly in the shoots. These complexes confer salt tolerance by enhancing SOS1 transport activity (Quan et al., 2007; Lin et al., 2009; Yang and Guo, 2018a). Furthermore, the *cbl8* mutant exhibits hypersensitivity to severe salt stress, highlighting the importance of *CBL8* in salt stress responses (Steinhorst et al., 2022). CBL5, an ortholog of CBL4 and CBL10 in *Arabidopsis*, interacts with and recruits CIPK8 and CIPK24 to the plasma membrane. This interaction is essential during seed germination, where CBL5 helps protect seeds and germinating seedlings from salt stress via the CBL5-CIPK8/CIPK24-SOS1 pathway (Xie et al., 2024). Another member of the CIPK family, CIPK8, forms a complex with SCaBP8/CBL10 and SOS1, promoting salt tolerance (Yin et al., 2020).

Numerous regulatory factors fine-tune the SOS signaling pathway, enhancing plant responses to salt stress (Ali et al., 2023). In parallel with the regulation at the post-translational level, several protein complexes have been identified recently that regulate the SOS pathway core components transcriptionally. Among them, the histone linker protein HIS1-3 (negatively) and the transcription factor WRKY1 (positively) are two proteins that regulate all three core elements of the SOS pathway. HIS1-3 and WRKY1 bind to the same loci on the chromatin of the SOS pathway genes and regulate their transcription (Wu et al., 2022). Short Root in Salt Medium1(RSA1)-RSA1 Interacting Transcription Factor1(RITF1) is another complex that positively regulates the SOS1 transcript through a nuclear calcium signaling pathway (Shi et al., 2003; Guan et al., 2013). MPK4-MYB42 is a module that activates SOS2 transcription in a UBC1/UBC2-dependent manner (Sun Y. et al., 2020; Zarreen et al., 2022). The transcription factor plant AT-rich sequence and zinc-binding protein 2 (PLATZ2) suppresses SOS3/SCaBP8 transcription, thereby negatively regulating salt stress tolerance (Liu S. et al., 2020). Under normal conditions, ABI2 (Ohta et al., 2003), GIGANTEA (GI) (Kim et al., 2013), 14-3-3 proteins, and SOS2-like protein kinase 5 (PKS5) (Zhou et al., 2014; Yang et al., 2019c) interact with SOS2 to inhibit its kinase activity. The interaction between SOS2 and ABI2 suggests a potential link between the salt stress response and ABA signaling pathways. Under high-salt conditions, GI is degraded, releasing its inhibition of SOS2, which subsequently activates SOS2 to promote salt tolerance (Kim et al., 2013). PKS5-mediated phosphorylation of SOS2 enhances its interaction with 14-3-3 proteins, but during salt stress, elevated Ca²^+^ levels promote 14-3-3 binding to PKS5, inhibiting PKS5 kinase activity and weakening the interaction between 14-3-3 and SOS2, thereby activating SOS2 (Yang et al., 2019c). GRIK1 phosphorylates and promotes SOS2 activity (Barajas-Lopez et al., 2018), while VPS23A enhances the SOS2–SOS3 interaction, promoting SOS2 localization to the plasma membrane (PM) (Lou et al., 2020). The SOS signaling pathway has been shown to connect salt stress with plant hormone signaling pathways (Yu et al., 2020). SOS2 enhances salt tolerance by inhibiting the kinase activity of CONSTITUTIVE TRIPLE RESPONSE1 (CTR1) through phosphorylation at serine 87 (S87), thereby activating the ethylene signaling response (Li et al., 2024). In addition, AFP2, a plant-specific ABI5-binding protein, serves as a negative regulator in the ABA signaling pathway and plays a key role in salt tolerance during seed germination. *AFP2* mutations increase sensitivity to salt stress. SOS2 physically interacts with and stabilizes AFP2, promoting the degradation of ABI5, a transcription factor that negatively regulates seed germination under salt stress. These findings suggest a potential link between salt stress and the ABA signaling pathway, opening new possibilities for enhancing plant resilience to environmental challenges (Wang et al., 2024a). Under salt stress, SCaBP8 inhibits the activity and PM localization of two type-D protein phosphatases (PP2C D6/D7), thereby activating SOS1 (Fu et al., 2023). PAMP-induced secreted peptide 3 (PIP3) binds and activates receptor-like kinase 7 (RLK7), which in turn activates MPK3 and MPK6, enhancing SOS1 activity and promoting Na^+^ homeostasis via ethylene/ROS signaling (Yu et al., 2010; Li et al., 2014; Zhou et al., 2022a). Salt-induced cytosolic Ca²^+^ increases facilitate the activation of phospholipase D (PLD) at the PM, generating the lipid messenger phosphatidic acid (PA) (Wang et al., 2006; Do et al., 2019). PA accumulation promotes the localization and enhances the kinase activity of SOS2 and MPK6 at the PM, facilitating SOS1-mediated Na^+^ efflux (Yu et al., 2010; Li et al., 2023a).

Beyond *Arabidopsis*, the SOS pathway is conserved in rice (Martinez-Atienza et al., 2007; El Mahi et al., 2019), maize (Zhang et al., 2016a; Zhou et al., 2022b; Li et al., 2023a, b), wheat (Feki et al., 2011; Yang et al., 2024), soybean (Zhang et al., 2022b), and tomato (Wang et al., 2021; Hong et al., 2023). In summary, the SOS pathway and its regulatory factors play crucial roles in promoting Na^+^ efflux from the roots into the soil solution and enhancing salt tolerance in shoot tissues (Quan et al., 2007; Liang et al., 2024).

### Intracellular compartmentalization of Na^+^

Researchers have long recognized that Na^+^ compartmentalization serves as an effective mechanism for plants to mitigate Na^+^ toxicity under salt stress conditions. Excess Na^+^ absorbed by plant roots can be sequestered into vacuoles or transported to the aerial parts of the plant. The central vacuole is particularly well-suited for Na^+^ sequestration, as it reduces cytoplasmic Na^+^ accumulation. This sequestration offers several advantages: it decreases Na^+^ concentration in the cytoplasm, preventing transport from underground parts to aerial tissues, thereby protecting plants from salt-induced toxicity. Furthermore, Na^+^ accumulation in vacuoles can act as an osmoregulatory substance, reducing cell water potential and promoting water uptake under salt stress (Yang and Guo, 2018a). While the size of plant cells is finite, the process of sequestering Na^+^ into vacuoles is an effective cellular strategy throughout the plant’s life cycle, safeguarding against Na^+^ toxicity caused by salt stress.

The sequestration of Na^+^ in vacuoles is mediated by vacuolar Na^+^/H^+^ exchangers (NHXs), which rely on the H^+^ gradient established by vacuolar H^+^-ATPases (V-ATPase, VHA) and H^+^-pyrophosphatases (V-PPase, VP) (Drozdowicz and Rea, 2001; Brini and Masmoudi, 2012). The NHX gene family is conserved across various plant species, including *Arabidopsis*, tomato, and several crops (Apse et al., 1999; Zhang and Blumwald, 2001; Zhang et al., 2001; Zhang and Shi, 2013). In *Arabidopsis*, the NHX family comprises six members, and overexpression of the *AtNHX1* gene has been shown to enhance salt tolerance in multiple plant species (Zhang and Shi, 2013). Studies indicate that the C-terminal region of AtNHX1 interacts with calmodulin (CaM) within the vacuole, inhibiting the transport activity of AtNHX1 in a Ca²^+^-dependent manner (Yamaguchi et al., 2003, 2005). Gain-of-function mutations in AtNHX1 can suppress salt hypersensitivity in *sos1* mutants by limiting Na^+^ accumulation in the cytoplasm and its transport to the shoots (Pabuayon et al., 2021). Additionally, AtNHX1 and AtNHX2 are instrumental in K^+^ accumulation in vacuoles and the regulation of vacuolar pH (Bassil et al., 2011b; Barragan et al., 2012). AtNHX5 and AtNHX6, localized in the Golgi, trans-Golgi vesicles, and prevacuolar compartments, are critical for maintaining the pH of these compartments (Bassil et al., 2011a; Reguera et al., 2015). The *nhx5 nhx6* double mutants exhibit salt hypersensitivity, likely due to the mislocalization of vacuolar transporters essential for Na^+^ sequestration (Bassil et al., 2011b).

In addition to NHX proteins, the vacuolar membrane-localized SCaBP8 also plays a pivotal role in Na^+^ sequestration by interacting with SOS2 (Kim et al., 2007; Yang et al., 2019b). Moreover, salt-induced endocytosis contributes to Na^+^ accumulation in vacuoles. For example, overexpression of *AtRab7*, a gene involved in vesicle trafficking regulation, enhances endocytosis in protoplasts, roots, and leaves, promoting Na^+^ accumulation in vacuoles (Mazel et al., 2004; Hamaji et al., 2009; Golani et al., 2013). In summary, NHX-type Na^+^/H^+^ antiporters are essential for mediating Na^+^ sequestration in plant cells, thereby promoting salt tolerance, growth, and development (**Figure 2**) (Zhang and Shi, 2013). However, the membrane transport mechanisms involved in intracellular Na^+^ sequestration remain poorly understood and warrant further investigation (Liang et al., 2024).

### Transporters of K^+^ and Cl^−^

In addition to Na^+^, maintaining K^+^ homeostasis under high salinity conditions is equally crucial for plant growth and development. Achieving K^+^ homeostasis in saline environments necessitates facilitating K^+^ influx while inhibiting K^+^ efflux (Yang and Guo, 2018a, b). K^+^ influx primarily relies on transporters such as HAKs and AKT (Gierth et al., 2005). In *Arabidopsis*, the HAK5 protein promotes high-affinity K^+^ uptake induced by K^+^ deprivation (Ragel et al., 2015). Similarly, in rice, OsHAK1, OsHAK5, OsHAK16, and OsHAK21 function analogously to *Arabidopsis* HAK5, enhancing K^+^ uptake under salt stress (Song et al., 2021). The AKT1 transporter is responsible for primary K^+^ uptake in *Arabidopsis* roots (Lagarde et al., 1996). Under salt stress, SOS2 promotes the phosphorylation of SCaBP8, alleviating its inhibitory effect on AKT1 and enhancing K^+^ uptake in the roots (Li et al., 2023). Conversely, the ZmHKT2 protein in maize negatively regulates K^+^ accumulation in the stems, which reduces the salt tolerance of maize (Cao et al., 2019). TaHKT9-B is a K^+^-preferring HKT transporter. The tae-miR390/TaTAS3/TaARF4/TaHKT9-B module has been identified as a crucial regulatory pathway in wheat under salt stress, offering valuable genetic resources for breeders aiming to enhance wheat salt tolerance (Du et al., 2024). These findings underscore the significance of maintaining K^+^ homeostasis for plant salt tolerance, emphasizing the need to study K^+^ transport mechanisms to improve crop salt tolerance.

Excessive Cl^−^ accumulation under salt stress can limit NO_3_^−^ uptake, transport, and assimilation (Li et al., 2017), potentially leading to Cl^−^ toxicity (Ren et al., 2021). Consequently, Cl^−^ uptake and transport processes are intricately linked to plant salt tolerance. Reported transporters include the *Arabidopsis* nitrate transporter/peptide transporters (AtNPF2.4 and AtNPF2.5), slow-type anion channel associated 1 homologs (AtSLAH1 and AtSLAH3), cation/Cl^−^ cotransporters (AtCLCc and AtCLCg), aluminum-activated malate transporter 9 (ALMT9), and the rice MATE (multidrug and toxic compound extrusion family) transporter BIG RICE GRAIN 1 (BIRG1). Additionally, ZmMATE29, type-A response regulator (ZmRR1), and histidine phosphotransfer protein 2 (ZmHP2) in maize maintain Cl^−^ homeostasis by facilitating Cl^−^ retrieval from the root xylem, vacuolar Cl^−^ accumulation, or Cl^−^ efflux from the roots, thereby regulating plant salt tolerance (**Figure 2**) (Colmenero-Flores et al., 2007; Negi et al., 2008; Jossier et al., 2010; Teakle and Tyerman, 2010; De Angeli et al., 2013; Baetz et al., 2016; Cubero-Font et al., 2016; Li et al., 2016a, b; Henderson et al., 2018; Ren et al., 2021; Yin et al., 2023).

## Salt stress and oxidative stress response

Throughout their life cycle, plants are constantly subjected to both biotic and abiotic stresses, which often lead to the accumulation of ROS and trigger stress responses, with salt stress being a significant factor. The generation of ROS in plants primarily involves four forms: hydroxyl radicals, hydrogen peroxide (H_2_O_2_), superoxide anions, and singlet oxygen (Yang et al., 2023; Jiang et al., 2024). Salt stress induces the transcription of genes encoding Respiratory Burst Oxidase Homologs D (RBOHD) and RBOHF, which subsequently catalyze the production of H_2_O_2_ (Miller et al., 2010). This process also triggers changes in Ca²^+^ signaling, mediating the overall response of plants to local salt stress through the RBOHD–RBOHF–H_2_O_2_–Ca²^+^ coupled signaling network (Evans et al., 2016). Excessive ROS accumulation can damage cells, leading to lipid peroxidation of cell membranes, DNA damage, protein denaturation, carbohydrate oxidation, pigment decomposition, and impaired enzyme activity (Noctor and Foyer, 1998).

To cope with elevated ROS levels induced by stress, plants synthesize antioxidant enzymes and non-enzymatic antioxidants to maintain ROS homeostasis. Antioxidant enzymes include superoxide dismutase (SOD), catalase (CAT), ascorbic acid peroxidase (APX), glutathione peroxidase (GPX), glutathione reductase (GR), dehydroascorbate reductase (DHAR), monodehydroascorbate reductase (MDHAR), and glutathione S-transferase (GST) (Das and Roychoudhury, 2014). Non-enzymatic antioxidants comprise glutathione, ascorbic acid, flavonoids, carotenoids, phenolic compounds, and tocopherols (Yang and Guo, 2018a). In high-salt environments, maize enhances salt tolerance by inducing ROS accumulation and activating the synthesis of various antioxidant enzymes through moderate expression of the male sterility gene *Open Reading Frame 355* (*ZmORF355*) (Xiao S. et al., 2023). Salt stress-induced ROS accumulation inhibits the accumulation of maize microRNA *ZmmiR169q*, promoting the transcription of the *antioxidant enzyme gene peroxidase 1* (*ZmPER1*), which mediates ROS elimination and protects against salt stress (Xing et al., 2022). Stress-induced ROS also inhibits the accumulation of maize *miR408*, increasing the transcription levels of its target genes *LACCASE 9* (*ZmLAC9*) and *ZmLAC18*, thereby promoting cell wall development and salt tolerance by regulating the polymerization of lignin monomers (Qin et al., 2023). *ZmIAA9*, a member of the maize *Aux/IAA* gene family, acts as a positive regulator of salt tolerance in maize, accompanied by increased ROS detoxification and elevated expression of ROS-scavenging genes. The transcription factor ZmbHLH32, part of the bHLH family, directly binds to the promoter region of ZmIAA9, activating its expression. The ZmbHLH32-ZmIAA9-ZmARF1 module is therefore crucial in regulating salt tolerance in maize (Yan Z. et al., 2023). Overexpression of rice SALT TOLERANCE RECEPTOR-LIKE CYTOPLASMIC KINASE 1 (OsSTRK1) leads to phosphorylation and activation of CATALASE C (CatC), resulting in higher CAT activity, reduced H_2_O_2_ accumulation, and enhanced salt tolerance compared to controls (Zhou et al., 2018). Transgenic rice lines expressing MIM396 and OE-*GRF6* show reduced H_2_O_2_ levels and increased activities of ROS-scavenging enzymes (CAT, SOD, and POD), contributing to significant enhancement of salt tolerance mediated by the miR396b/GRF6 module (Yuan et al., 2024). The rice OsTET5 (tetraspanins) protein regulates reactive oxygen species homeostasis by modulating the expression and activity of antioxidant pathway enzyme genes, as well as the accumulation of proline. This regulation contributes to enhancing the salt tolerance of rice (Mani et al., 2024). OsNF-YC5 encodes a putative subunit of the NF-Y transcription factor in rice. The osnf-yc5 mutant exhibits reduced levels of H_2_O_2_ and malondialdehyde (MDA), as well as increased CAT activity under salt stress. Furthermore, in the mutant lines, both ABA-dependent marker genes (OsABI2 and OsLEA3) and ABA-independent marker genes (OsDREB1A, OsDREB1B, and OsDREB2A) are upregulated in response to salt stress. These results suggest that knocking out OsNF-YC5 enhances rice salt tolerance by boosting CAT enzyme activity and modulating gene expression in both ABA-dependent and ABA-independent pathways (Yan et al., 2024). Furthermore, ABA demonstrates a notable role in managing salt stress-induced oxidative stress in salt-sensitive rice cultivars. Under salt stress conditions, ABA-treated Swarna sub1 (a salt-sensitive rice variety) exhibits increased relative water content, an elevated K^+^/Na^+^ ratio, and enhanced cell membrane stability. Additionally, ABA treatment reduces cell wall peroxidation, leading to the enlargement of the endodermal lumen and a decrease in malondialdehyde content. In summary, rice varieties with higher accumulation of ABA can improve their salt tolerance (Deng et al., 2023; Das et al., 2024). The mutation in the gene encoding a cytochrome b561 domain-containing protein (OsCYBDOMG1) results in decreased ascorbic acid (AsA) content and AsA/DHA (dehydroascorbate) ratio, leading to increased H_2_O_2_ accumulation and reduced salt stress tolerance (Deng et al., 2023). Salt stress induces the expression of GDP-mannose pyrophosphorylase (vitamin C1, VTC1), promoting ascorbic acid (AsA) synthesis and salt tolerance in *A. thaliana* (Zhang et al., 2012). RADICAL-INDUCED CELL DEATH 1 (RCD1) is identified as an essential player in salt stress response. *Arabidopsis* NAC domain-containing protein 17 (ANAC017) functions downstream of RCD1 in the salt stress response and plays a negative role by impairing SOD enzyme activity. RCD1 promotes salt stress response and maintains ROS homeostasis by inhibiting the activity of ANAC017 (Tao et al., 2023). The wheat WRKY transcription factor TaWRKY17 enhances salt stress tolerance by regulating ABA/ROS-related and stress-responsive genes, as well as by increasing antioxidative stress capabilities. Overexpression of *TaWRKY17* significantly improves the plant’s tolerance to salt stress. Under salt stress conditions, compared to the wild type (WT), transgenic wheat plants overexpressing *TaWRKY17* exhibit increased enzyme activities of SOD, POD, and CAT, while the accumulation of H_2_O_2_ is reduced. Furthermore, the transgenic wheat plants show regulated expression of ABA/ROS-related and stress-responsive genes, leading to enhanced tolerance to salt stress (Yu Y. et al., 2023). Under salt stress conditions, the levels of SOD and proline in wheat overexpressing *TaGB1-B* (G-Protein β-Subunit Gene) were higher than those in the control, while the concentration of MDA was lower. This indicates that TaGB1-B enhances the salt tolerance of wheat by scavenging ROS (Xiong et al., 2023). The overexpression of the wheat 2-Cys peroxiredoxin gene TaBAS1 enhances tolerance to oxidative stress by promoting the activity of ROS-scavenging enzymes and reducing the accumulation of ROS under salt stress, thereby enhancing salt tolerance at both the germination and seedling stages of wheat (Xiao G. et al., 2023). Furthermore, under salt stress conditions, the ectopic expression of wheat BR synthesis gene *TaDWF4* and BR signaling gene *TaBAK1* can enhance plant salt tolerance by balancing the levels of ROS in the roots (Hou et al., 2024). Through these enzymatic and non-enzymatic reactions, plants maintain ROS stability to cope with various abiotic stresses, including salt stress (Zhang et al., 2014, 2017; Wang et al., 2020d). Additionally, the salt-induced ABA signaling pathway regulates the production and distribution of ROS within plants, mitigating damage caused by stress-induced ROS accumulation and enhancing plant salt tolerance (Zhang et al., 2009; He et al., 2022).

## Salt stress and stem cell development

Pluripotent stem cells play a pivotal role throughout the entire life cycle of biological development, governing the continuous regeneration of tissues and organs. This sustained regenerative capacity enables many plants to survive for hundreds or even thousands of years (Sang et al., 2018; McKim, 2019; Hata and Kyozuka, 2021; Umeda et al., 2021). The localization of stem cells within specific microenvironments is crucial for cellular differentiation, allowing these initially undifferentiated cells to retain robust self-renewal capabilities (Aichinger et al., 2012; Wang et al., 2020b; Nicolas and Laufs, 2022). In recent years, stem cell research has primarily focused on elucidating the processes of stem cell initiation, maintenance, and signal transduction (Sablowski, 2011; Janocha and Lohmann, 2018; Motte et al., 2019; Fujinami et al., 2020; Xue et al., 2020). As sessile organisms, plants constantly face challenges from abiotic stresses throughout their life cycle, including drought, salinity, temperature fluctuations, heavy metal ion exposure, and ultraviolet radiation (Sun H. et al., 2020; Markham and Greenham, 2021; Zhang et al., 2022a; Kopecka et al., 2023).

Elevated soil salinity affects plant development, inevitably leading to reduced crop yields. The SOS signaling pathway serves as a conserved and vital regulatory mechanism for excluding Na^+^ and mitigating its toxic long-distance transport (Shi et al., 2002; El Mahi et al., 2019). This pathway involves Ca²^+^ sensors, kinases, and Na^+^/H^+^ exchanger modules. Salt stress induces the expression of *GSO1* in the endodermis and meristems (Chen et al., 2023; Jiang et al., 2024). The GSO1–SOS2–SOS1 module functions as a dedicated intracellular Na^+^ detoxification channel, enabling roots to continue growing in high-salt environments (Chen et al., 2023). The plant transcription factors PLETHORA1/2 (PLT1/2), featuring the AP2 domain, play a pivotal role in regulating responses to salt stress. Under salt stress conditions, the SOS2 protein phosphorylates PLT1/2, stabilizing them and thereby preserving the activity of meristematic tissues, which facilitates the recovery of root growth once the salt stress abates (Hao et al., 2023).

**Figure 3:**
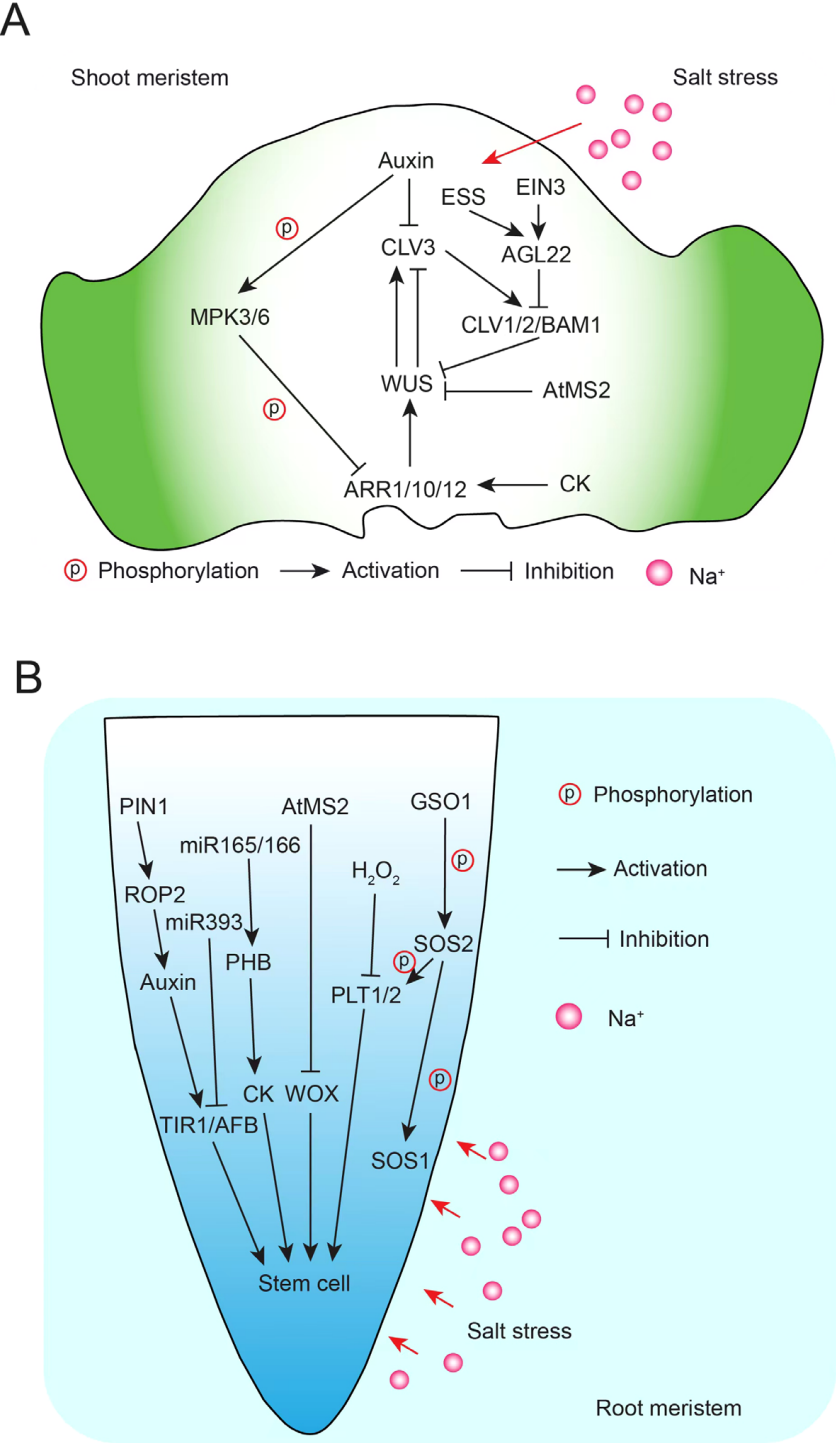
A schematic diagram of stem cell development and salt stress. **(A)** Signaling pathways of shoot meristem stem cell development under salt stress. **(B)** Signaling pathways of root meristem stem cell development under salt stress.

Simultaneously, the resilience and adaptability of apical meristem stem cells to salt stress are closely tied to redox reactions, ROS, nitric oxide (NO), microRNAs, and plant hormones such as auxin and cytokinins (Yang and Lee, 2023; Yu et al., 2020). Plant hormones are crucial for regulating responses to salt stress, which typically impedes plant growth. Research has shown that stress hormones, including ABA, SA, JA, and ethylene, as well as growth hormones like auxin, cytokinins (CKs), gibberellins (GAs), and brassinosteroids (BRs), play vital roles in mediating salt stress signaling while balancing growth and stress responses. Some hormones positively influence salt tolerance, while others can have inhibitory effects (Yu et al., 2020; Jiang et al., 2024). Salt stress has been associated with altered redox status in *Arabidopsis* root meristems, affecting root growth (Jiang et al., 2016). Under optimal conditions, the region with the lowest redox potential in the quiescent center (QC) coincides with the area of maximum auxin accumulation (Jiang et al., 2016; Smolko et al., 2021). However, under salt stress, auxin signaling in this region is notably reduced (Smolko et al., 2021). Auxin is known to support developmental plasticity under abiotic stresses, including salinity, and promotes ROS production via NADPH oxidase activation (Bartoli et al., 2013; Korver et al., 2018). These findings suggest a link between redox balance and auxin regulation under salt stress, collectively influencing adaptive root growth. Salt stress also induces miR393 expression, which suppresses auxin receptor proteins, such as TRANSPORT INHIBITOR RESISTANT1 (TIR1) and AUXIN SIGNALING F-BOX (AFB), at the post-transcriptional level (Iglesias et al., 2014). In *mir393ab* and *tir1 afb2* mutants, the inhibitory effect of salt on root growth is less pronounced compared to WT plants (Iglesias et al., 2010, 2014). This suggests a complex network of salt-responsive miRNAs, redox status, and auxin dynamics in the root meristem, which is crucial for root plasticity under salt stress. NO also plays a significant role in plant growth and development, particularly in the root system (Ma et al., 2020). Salt stress-induced NO signaling reduces cell division and promotes cell differentiation in the root meristem (Fernandez-Marcos et al., 2011). Moreover, using NO biosynthesis inhibitors can mitigate the salt-induced inhibition of root meristem growth (Liu et al., 2015). The protein PIN1, which regulates auxin distribution, modulates Rho-of-plant 2 (ROP2) GTPase-mediated endocytic recycling in the root meristem, essential for NO-mediated root growth inhibition (Kenesi et al., 2023). Additionally, salt stress decreases the expression of miR165 and miR166, leading to an upregulation of PHABULOSA (PHB) and increased production of cytokinins, which are associated with pre-differentiation signaling in root meristems (**Figure 3**) (Scintu et al., 2023).

Stem cell homeostasis in the shoot apical meristem (SAM) is primarily governed by a feedback loop between CLAVATA3 (CLV3) and WUSCHEL (WUS) (Kitagawa and Jackson, 2019). Interestingly, CLV3 loss-of-function mutants, which enhance stem cell signaling in the SAM, exhibit a salt-tolerant phenotype in overall stem growth compared to WT plants under salt stress (Jun et al., 2019). Additionally, double mutants with loss-of-function in CLV1 and BAM1, receptors for the CLV3 peptide in the SAM, show higher survival rates under salt stress (Jun et al., 2019; Lee et al., 2019). These findings suggest that CLV3p-CLV1/BAM1 signaling may contribute to stress tolerance, including salinity. The SAM’s activity, indicated by cell division in *clv3-2* mutants, appears essential for this salt tolerance effect. Moreover, MPK3 and MPK6 are implicated in the salt stress response. Under salt stress, these kinases phosphorylate and degrade *Arabidopsis* Response Regulators ARR1, ARR10, and ARR12, enhancing stress tolerance (Yan Z. et al., 2021). ARR proteins are central to CK signaling, integral to plant development and stem cell maintenance (Hwang et al., 2012). CK signaling has been shown to inhibit growth adaptation under high-salt conditions (Nishiyama et al., 2011). In addition, the MKK7–MPK6 module modulates SAM growth, as constitutive MKK7 expression leads to SAM defects (Doczi et al., 2019), underscoring the probable importance of MAPK signaling in salt-induced stress adaptation in the SAM. Proline, an osmolyte and ROS scavenger, accumulates in many plant species under salt-induced osmotic stress (Verslues and Sharma, 2010; Meena et al., 2019). Proline-mediated regulation bridges salt stress and SAM plasticity, aiding SAM growth adaptation (Mattioli et al., 2008; Szekely et al., 2008; Mattioli et al., 2009, 2022). Similar to root plasticity, the redox balance maintained by ROS is crucial for regulating SAM development under salt stress by managing the balance between stem cell proliferation and differentiation (Lee, 2018). Endogenous stress-related signals (ESS), including stress hormones, regulate stem cell maintenance in the SAM under natural growth conditions. Ethylene signaling, mediated by the transcription factor EIN3, activates AGAMOUS-LIKE 22 (AGL22), which represses CLV1 and CLV2 and is a key regulator in stress-responsive gene networks (Gregis et al., 2013; Bechtold et al., 2016). AGL22 thus functions as a signaling hub for the SAM’s developmental plasticity in response to ESS and external stress like salinity (Zeng et al., 2021). Recent studies have also highlighted the role of the prion-like domain (PrD) in the SAM regulator SHOOT MERISTEMLESS (STM), which forms nuclear condensates under salt stress, enhancing plant salt tolerance (Cao et al., 2023). A combined metabolomic and transcriptomic analysis of CK signaling-deficient *Arabidopsis* mutants (*ahp2,3,5* and *arr1,10,12*) under salt stress demonstrated that CK signaling reprograms gene-metabolic networks linked to salinity responses (Abdelrahman et al., 2021). Furthermore, *Arabidopsis* methionine synthase 2 (AtMS2) inhibits stem cell maintenance under salt stress. While primarily cytoplasmic under normal conditions, AtMS2 accumulates in the nucleus under salt stress, where it interacts with WUS/WOX proteins to repress WUS/WOX expression, thus limiting stem cell maintenance. Mutations in AtMS2 result in increased salt tolerance, indicating that AtMS2 acts as a negative regulator of stem cell maintenance under stress (Qiu et al., 2024). Collectively, these findings suggest intricate interactions between salt stress signaling and the regulatory pathways governing meristem stem cell homeostasis (**Figure 3**) (Yang and Lee, 2023).

## Conclusion and perspectives: improvement of crop salt tolerance

In recent years, global climate change has intensified, leading to more frequent and prolonged environmental stress events that pose significant challenges to plant growth, development, and crop yield. As sessile organisms, plants have evolved intricate systems to withstand abiotic stresses. This article reviews the findings of researchers on salt stress in plants such as *A. thaliana* and important crops including wheat, maize, and rice. It summarizes key issues such as how plants perceive salt (Na^+^) stress; the mechanisms of response; Na^+^ transport, compartmentalization, and clearance; and changes in ROS induced by salt stress. Furthermore, researchers have explored the regulation of plant stem cell development by salt stress and the interplay between plant hormones and salt stress regulation. Meanwhile, we also summarize some genes and protein factors involved in the above biological processes (**Supplementary Table S1**). Additionally, there has been a growing focus on the advantages of salt stress proteins and rhizosphere microbes in enhancing plant salt tolerance and increasing crop yields under high-salinity conditions (Athar et al., 2022; Liu et al., 2022c).

However, many questions and challenges persist regarding how plants, especially crops, cope with salt stress and how we can apply our understanding of plant salt tolerance to boost food crop yields in saline–alkali soils while developing new salt-tolerant varieties (Liang et al., 2024). Currently, advancements in single-cell and spatial transcriptomics, as well as high-throughput phenomics platforms, have facilitated a comprehensive understanding of plant responses to salt stress at the molecular, cellular, and subcellular levels. Quantitative trait locus (QTL) mapping and genome-wide association studies (GWAS) have gradually identified crucial abiotic stress response regulators and natural allelic variations in crop species. Integrated analyses of transcriptomics, spatial transcriptomics, proteomics, metabolomics, and phenomics provide effective and rapid tools for mining crop salt tolerance genes and screening for high-quality salt-tolerant lines. The development of various gene-editing technologies will expedite the utilization of salt tolerance genes identified through forward or reverse genetics in breeding new salt-tolerant varieties. Combining big data-based artificial intelligence (AI) with foundational knowledge of plant salt tolerance will enable the simulation and prediction of crop responses to salt stress, facilitating the molecular design of salt-tolerant crops and the breeding of new varieties that are both salt-tolerant and high-yielding.

Given the global importance of wheat, maize, and rice for food security, conducting salt tolerance research on these crops and breeding salt-tolerant, high-yield varieties are invaluable for optimizing the utilization of saline–alkali soils and ensuring a stable food supply. With continuous advancements in scientific and technological methods, the mechanisms underlying crop salt tolerance are gradually being unraveled. These studies are expected to contribute to the cultivation of more salt-tolerant crop varieties and promote the development of high and stable crop yields in high-salinity environments.
